# Body-mass index and obesity in infertile couples in southwest China

**DOI:** 10.1097/MD.0000000000036494

**Published:** 2023-12-15

**Authors:** Yutao Li, Ke Dou, Qun Lv, Yuan Wu

**Affiliations:** a Department of assisted reproduction center[aff_start], [/aff_end]Sichuan Academy of Medical Science & Sichuan Provincial People’s Hospital, University of Electronic Science and Technology of China, Chengdu, Sichuan, China; b Department of urology[aff_start], [/aff_end]Sichuan Academy of Medical Science & Sichuan Provincial People’s Hospital, University of Electronic Science and Technology of China, Chengdu, Sichuan, China.

**Keywords:** adults, body mass index, infertility, obesity, socioeconomic characteristics

## Abstract

To investigate body mass index (BMI) of infertile couples and analyze its related influencing factors in Southwest China, so as to prevent and control the obesity. We analyzed the data of a total number of 8877 infertile couples who received treatment in our assisted reproductive center from October 2012 to March 2022. The mean age and BMI of men and women were 33.5 years, 23.9 kg/m^2^ and 31.6 years, 21.9 kg/m^2^. The prevalence of overweight (BMI 25–29.9) was 30.9% in men and 14.7% in women, 3.7% of men and 1.6% of women were obese (BMI ≥ 30), while 3.6% of men and 10.8% of women were underweight (BMI<18.5). Multivariable linear regression analysis indicated that the age and educational background of both women and men had an impact on BMI. In our study, the proportion of male obesity and overweight is much higher than that of female. On the other hand, the proportion of females with low weight was higher than that of males. The age and educational background of men and women have a certain correlation with BMI.

## 1. Introduction

With the development of society and the popularization of mechanization, people are engaging in fewer physical activities. The prevalence of obesity in China has significantly increased over the years. According to a study conducted in 2014, China ranked second in terms of obesity rates for both men and women, compared to 60th place for men and 41st place for women in 1975.^[1]^ Another recent study with a large sample size from China found that the body-mass index (BMI) levels rose from 22·7 kg/m² in 2004 to 24·4 kg/m² in 2018 and obesity prevalence from 3.1% to 8.1%.^[2]^

BMI is considered a reasonably good measure of overall adiposity.^[3]^ There are many factors that affect BMI, such as age, physical activity, eating habits and so on.^[4,5]^ However, due to differences in ethnic, religious, economic and cultural beliefs, the factors affecting BMI also present certain localized characteristics.^[6]^ According to the World Health Organization, women tend to have higher obesity rates compared to men. One of the primary contributing factors is the significant increase in BMI that occurs during pregnancy and childbirth.^[7]^

Accordingly, in order to eliminate the influence of pregnancy and childbirth on women BMI, we used data from 8877 infertile couples who received treatment at our assisted reproductive center from October 2012 to March 2022, to systematically assess the association between BMI and demographic and socioeconomic characteristics. As far as we know, no previous studies have investigated this association in infertile couples. Therefore, acquiring this knowledge could suggest ways to target interventions and provide future health care requirements.

## 2. Materials and methods

### 2.1. Study design and subjects

This retrospective analysis was based on the data of 8877 couples presenting for evaluation of infertility at our assisted reproductive center from October 2012 to March 2022. The criteria for inclusion in the study were a history of infertility for at least 1 year, the female partners aged 20 to 56 years, and male partners aged 22 to 60 years. This project was submitted to the Research Ethical Committee of Sichuan Academy of Medical Science & Sichuan Provincial People Hospital (Case number, 20230104), from the principles of ethical and legal aspects that govern scientific research on human beings, and the principles expressed in the Declaration of Helsinki.

Participants were inquired about their current age, race, level of education, and duration of infertility. A total of 8877 infertile couples’ data were analyze. Weight and height measurements were obtained using a digital self-calibration scale, with weight recorded to the nearest 0.5 kg and height to the nearest 0.5 cm. BMI was calculated as kg/m^2^, and extreme values (BMI<12 kg/m^2^ or BMI>50 kg/m^2^) were excluded. BMI categories recommended by the World Health Organization were used: underweight<18.5 kg/m^2^, normal weight 18.5 to 24.9 kg/m^2^, overweight 25.0 to 29.9 kg/m^2^, obese ≥ 30 kg/m^2^.^[8]^ The population analyzed in this study mainly consisted of individuals from southwest China, where eating habits are relatively uniform. Due to the difficulty of quantifying physical activity, 2 main variables associated with BMI, namely diet and physical activity, were not included. Three independent variables were used for analysis: age (20–29, 30–39, 40–49, ≥50), race (Chinese Han, Chinese Zang, Chinese Yi, other ethnic groups) and education level (<high school, high school,> high school).

### 2.2. Statistical analysis

Descriptive statistics, such as frequency, percentage, mean, median, minimum, maximum value and standard deviation were used in this study. Multiple linear regression was used to analyze the associations between demographic, socioeconomic characteristics and BMI of men and women. All tests were 2-sided and a *P* value of ≤ .05 was considered statistically significant. All calculations were performed on Spss 27.0.

## 3. Results

**Figure 1.**
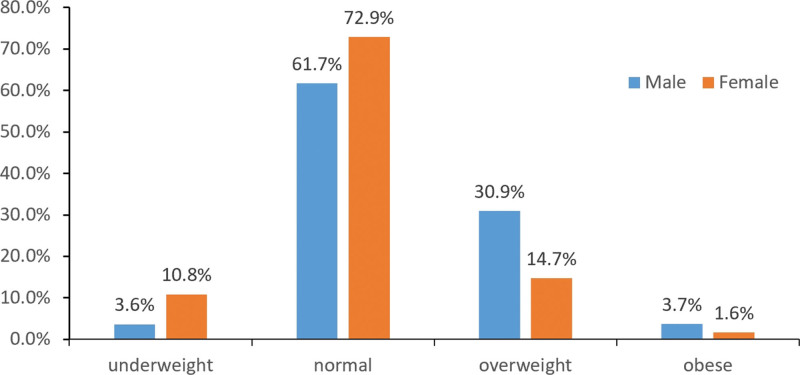
The BMI distribution of the infertile couples. BMI = body mass index.

A total of 8877 couples were included in the analysis. The mean BMI of men and women was 23.9 kg/m^2^ and 21.9 kg/m^2^, respectively. Figure 1 presents the prevalence of being underweight (BMI < 18.5), normal weight (BMI 18.5–24.9), overweight (BMI 25–29.9) and obese (BMI ≥ 30) by gender; Among the men, 61.7% were of normal weight, while among the women, 72.9% were of normal weight. Furthermore, 30.9% of men and 14.7% of women were overweight, and 3.7% of men and 1.6% of women were obese. Additionally, 3.6% of men and 10.8% of women were underweight (Fig. 1).

Subgroup analysis showed that the variables of age, education and nationality, were significantly associated with BMI. In both men and women, older age was predictive of increased BMI. The relationship between education level and BMI differed between genders, with a positive correlation in men and a negative correlation in women. Among the ethnic groups, Chinese Han men and the other ethnic had a lower BMI compared to Zang and Yi ethnic groups (*P* < .05), while there was no significant difference between the Zang and Yi ethnic groups or between the Han and other ethnic groups. There were no statistical difference in BMI of different ethnic groups among women (Table 1). Multivariable linear regression analysis indicated that both age and educational background had an impact on BMI in both women and men (Table 2).

**Table 1:** Association of couples’ demographic characteristics with frequency of sexual intercourse.

Characteristic	N*	%	Mean(SE)	*P* value
Man age (yr)				
20–29	2046	23.1%	23.26(0.07)	<.01
30–39	5604	63.1%	23.95(0.04)	
≥40	1227	13.8%	24.50(0.08)	
Woman age (yr)				
20–29	3237	36.5%	21.63(0.06)	<.01
30–39	4989	56.2%	21.97(0.04)	
≥40	651	7.3%	22.78(0.11)	
Man education				
<High School	2184	25.1%	23.7 (0.07)	<.01
High School	3645	41.9%	23.8 (0.05)	
>High School	2874	33.0%	24.1 (0.05)	
Woman education				
<High School	2349	26.9%	22.6 (0.07)	<.01
High School	3951	45.2%	21.8 (0.05)	
>High School	2432	27.9%	21.5 (0.06)	
Man nationality				
Han nationality	8343	94.1%	23.8 (0.03)	<.01
Zang nationality	246	2.8%	25.2 (0.20)	
Yi nationality	95	1.1%	24.5 (0.35)	
other nationality	183	2.0%	23.9 (0.23)	
Woman nationality				
Han nationality	8299	93.6%	22.2 (0.03)	.08
Zang nationality	242	2.7%	22.5 (0.18)	
Yi nationality	106	1.2%	22.7 (0.30)	
other nationality	221	2.5%	22.1 (0.21)	

* Numbers may not tally due to missing information.

**Table 2:** Multivariable linear regression analysis examining BMI among infertile couples (N = 8877).

Model	Unstandardized coefficients B	Std. error	Standardized coefficients beta	t	*P*	95.0% confidence interval for B
(Constant)	18.217	0.237		76.767	<.001	17.752~18.682
Woman education	0.617	0.044	0.147	13.938	<.001	0.53~0.703
Woman age	0.078	0.007	0.124	11.761	<.001	0.065~0.091
Man age	0.077	0.006	0.130	12.172	<.001	0.065~0.089
Man education	−0.127	0.045	−0.030	−2.829	.005	−0.214 to −0.039
Man ethnic	0.169	0.069	0.026	2.461	.014	0.034~0.303

## 4. Discussion

In this large retrospective study involving 8877 infertile couples aged 20 to 60 years, we found that men in southwest China have a higher prevalence of overweight and obesity compared to women. The prevalence of overweight was 30.9% in men and 14.7% in women; obesity was 3.7% in men and 1.6% in women, respectively. In contrast, women had a higher prevalence of underweight than men (3.6% men and 10.8% women). We also found that age and educational level were significant risk factors for overweight and obesity. Increasing age was the major responsible factors for higher BMI in both women and men, and low level of education was associated with lower BMI in men but higher BMI in women.

The results of the present study are somewhat different from the results reported from other regions of China. In a study conducted in Guizhou Province, Southwest China, focusing on Bouyei and Han Peoples aged 20 to 80 years, the prevalence of general obesity was 10.6% in Han people and 4.8% in Bouyei people.^[9]^ It is important to note that their criteria for diagnosing obesity (BMI ≥ 27.5) are lower than ours (BMI ≥ 30). In addition, although both of our research objects are in southwest China, the climate, geography and lifestyle of Guizhou, especially the Bouyei people, is very different from ours. These factors could explain observed difference in obesity prevalence between genders, ethnic groups, and regions. In another study of Chinese population living in Jiangxi Province, the prevalence rate of obesity and overweight were slightly higher than ours. In the latter study the respective prevalence rates of obesity and overweight in the male and female subjects were 25.9% and 25.7%; and 8.4% and 7.6%.^[10]^ In another earlier study of Chinese adult population living in 7 provinces in China during 1989 to 1997, Bell et al have reported the prevalence of overweight in men and women 20 to 45 years of age was 13.6% and 19.2% and for obesity 0.5% and 1.5%.^[11]^ In this study, the prevalence rates of overweight and obesity were lower compared to our findings. This discrepancy may be attributed to China relatively low economic development and comparatively lower living standards during the 1990s. Furthermore, a recent study from northern China reported that the average BMI for men and women was 23.9 kg/m^2^ and 21.9 kg/m^2^, which is higher than our finding.^[12]^ This could be due to the geographical environment, as historically nomadic populations in northern China tend to have better physical strength compared to farming communities in the south. Additionally, dietary differences play a role, with wheat, beef, and mutton being staples in the north, while rice and pork are more prevalent in the southern regions. These regional and dietary disparities contribute to the higher BMI observed in northern China compared to southern China.

In 2014, there were approximately 266 million obese men and 375 million obese women worldwide, with 58 million men and 126 million women being severely obese.^[1]^ A large proportion of the world obese adults lived in high-income developed countries. The prevalence of obesity in various studies from around Asia has shown considerable variation. In Asia, the prevalence rates of obesity reported from 5.9% to 26% for men and 8.5% to 44% for women.^[13–20]^ The prevalence of overweight and obesity in our study is lower than the values reported by these studies. The estimates of obesity prevalence are influenced by factors such as the definition of obesity used, methodological factors, and the demographic characteristics like age, ethnicity, and social class of the population sample investigated. Contrary to previous findings, our study revealed a higher prevalence of overweight and obesity among men compared to women.^[20–23]^ This outcome could be attributed to the exclusion of the effects of pregnancy and labor on women in our study. Excessive gestational weight gain and postpartum weight retention play an important role in long-term obesity in women of reproductive age.^[24,25]^

Based on the present study, age and education level have significant influence on the BMI of couples. The association between age and BMI as shown in our study was also observed by other studies.^[12,26,27]^ Age has been considered as a predicted factor for obesity in a series of published studies.^[28,29]^ This can be attributed to the gradual decrease in basal metabolic rate with increasing age, resulting in a higher prevalence of overweight individuals. The relationship between education level and BMI in this study is consistent with a previous study by Wang,^[2]^ but is slightly different from other studies.^[30–33]^ These studies have shown that low-educated individuals had higher rates of overweight and obesity. Interestingly, our study revealed a reversed effect of education on the BMI of men and women: men with higher education displayed higher BMIs, while the opposite trend was observed in women. One possible explanation for this finding is that men with higher education tend to be more involved in mentally demanding work, leading to insufficient physical activity and an increased risk of obesity. Conversely, regardless of education level, women are generally engaged in less physical work, and those with higher education tend to pay more attention to the management of their figure and weight. Therefore, a higher education level is associated with a lower incidence of obesity among women.

There were several strengths and limitations in our study. First, as a retrospective study, the current analysis was limited in its ability to elucidate causal relationships between risk factors and overweight. Moreover, BMI is not entirely accurate as it can overestimate body fat in individuals who are muscular and underestimate body fat in individuals who have lost muscle mass, such as older adults.^[34]^ Second, certain variables, including detailed dietary habits, family income, and physical activity, were not included in the analysis. Third, the results may have been influenced by recall bias since all information was self-reported. Finally, it is important to note that the participants were young and middle-aged people with infertility recruited from southwest China. Therefore, this conclusion may not be representative of other regions or the entire population of China.

## 5. Conclusion

In conclusion, this study described the epidemiology of overweight and obesity among infertility adults in southwest China and evaluated the association between age, sex, ethnicity, education level, and BMI. Due to the unique geography and characteristics of the individuals we include, the prevalence of obesity was relatively low. The results indicated that age and education level were significantly associated with BMI. As age increases, women with lower education levels and men with higher education levels should be given higher priority for obesity preventing and control.

## Acknowledgments

We thank all the couples for participating in the study. This research was supported by the key research and development project of Sichuan Province Science and Technology Department Foundation (grant 2023YFS0174).

## Author contributions

**Conceptualization:** Yutao Li.

**Data curation:** Yuan Wu, Yutao Li.

**Formal analysis:** Ke Dou.

**Investigation:** Yutao Li.

**Methodology:** Yuan Wu, Ke Dou.

**Project administration:** Qun Lv.

**Supervision:** Qun Lv.

**Writing – original draft:** Yutao Li.

**Writing – review & editing:** Ke Dou, Qun Lv.
